# Trichinæ and Trichinosis

**Published:** 1866-07

**Authors:** Ch. Eyrich

**Affiliations:** Newark, New Jersey


### SELECTIONS.

*Trichinæ and Trichinosis.* By CH. EYRICH, M. D., of Newark, New Jersey.

In consequence of the recent repeated occurrence of a peculiar disease, caused by the introduction into the human body of parasitical animals called *trichinæ*, this subject has again attracted the attention of the profession; and, although I have no observations of my own to offer, a review of some of the leading facts connected with the growth, development, and migration of *trichinæ*, and the disease termed *trichinosis*, will perhaps be of interest. My sources of information are derived chiefly from recent publications in German.

*Anatomy and Development of Trichinæ.*—At a meeting of the Society of Physicians of Vienna, January 19th, 1866,* Dr. Wedl spoke at length on the anatomy and development of trichinæ. The first discoverer of trichinæ was Hilton, of England, who found them imbedded in the muscles of a cadaver, but believed them to be ecchynococci. In 1835, Richard Owen found in some muscles a worm, rolled up cork-screw fashion, which, from this circumstance, he named *trichina spiralis.*

Regarding the sexual development of these animals, observers were for a long time in the dark. Siebold looked upon the muscular trichina as some other worm in an undeveloped state. Later, it was thought to be identical with trichostoma or filiaria. Virchow first recognized and described the true sexual development of this animal; Leuckart added to our knowledge of its anatomical relations, while Zenker, by his well-known case, shed light upon its pathological significance. Fuchs and Pagenstecher have since then contributed accurate anatomical descriptions of the animal.

It possesses an elongated cylindrical body, tapering toward the head, and thicker at the other extremity. This thread-like form has essentially caused it to be confounded with the filiariæ. Its skin is striped and curled. The digestive tube begins at the head, dilating in the centre into a sort of stomach, and terminating at the caudal extremity into an anus. At the anterior surface of the body there is an accumulation of cells, which might erroneously be taken for ovaries; more careful investigations have shown them,

* Medic. Neuigkeiten.

however, to be secretory organs, probably the sudorific glands of the animal; at the posterior surface, on a level with the stomach, there are also glanular organs, in which probably the secretion of digestive fluids takes place.

The female, about a third larger than the male, possesses near the extremity of the head a tube representing a vagina; this dilates into a uterus, terminating in ovaries. In the uterus the ova are found imbedded, containing the embryones in capsule, and where they are brought to maturity; the trichinæ hence are animals giving birth to live offspring. The male presents at the caudal extremity a common cloaca, which separates into rectum and penis. A further examination of this cloaca leads to traces of a vas deferens, the vesicula seminalis, and the testicle. Coition is accomplished by the male winding itself around the female, and evacuating the semen into the vagina of the female. The development of the embryo takes place in this manner: That the contents of the ovum, at first containing but one granulated nucleus, after fruition separates into yolky spheres. The embryo at first has a pear-shaped, subsequently a cylindrical form. After the living embryo has been discharged, the migration commences, and is particularly aided by the form of the animal. With its thin anterior extremity it makes perforating boring efforts, and in this manner it slides easily through the smallest interstices of the tissues. Commonly, the direction of travel is along the fibres of the areolar tissue. Exceptionally, it may happen that a trichina gets into the current of the blood, and is carried by it to remoter parts; as a rule, however, the muscles are reached in the manner first noticed.

Here a further metamorphosis takes place by its becoming surrounded with a capsule. Imbedded in this calcareous capsule, within the continuity of the muscular fibres, the animal continues to live and becomes sexually developed. If the incapsulated muscular trichina reaches the stomach, observation teaches that the calcareous capsule becomes dissolved and destroyed. But the animal, freed from its envelope, continues to live, propagates and give rise to trichinosis.

*Mode of Migration, etc.*—Dr. Furstenberg, Professor of Zoology, and author of an excellent treatise on the acarus scabiei, has experimented since 1863 on the manner in which the embryos of trichinæ travel to their ultimate habitation in the primitive bundles of the voluntary muscles. The following are the main points of his researches:

The intestinal walls are always perforated by the trichinæ embryones, in order to reach their place of destination. But they do not always perforate the three coats of the intestines. A part of them pierce only the mucous and muscular coat, and then move along the connective tissue of the two laminæ of the serous coat, which form the mesentery, upward toward the spinal column, thence continuing their migrations to the muscles. Those embryos which perforate all three coats reach the free surface of the peritoneum, whence they start on their further journey.

As long as the migration of the trichinæ from the guts to the muscles lasted, trichinæ embryos were always found in the free space of the abdominal cavity. They were never found absent during the first thirty days of feeding the animals experimented on with trichinous meat, which confirms Virchow's and Leukhart's theory of trichina migration. Neither in the chambers of the heart, nor in the blood-vessels, have embryos or fully developed trichinæ been found, if the examinations were conducted with proper care. In blood coagula Fuchs has sometimes found that they had originally been there and not got there by accident.

The occurrence of trichinæ in the mesenteric glands is easily explained from the circumstance that the embryos, as stated, travel frequently between the layers of the mesentery upward toward the spinal column; hence they need not first enter the lymphatics in order to reach the glands situate between the laminæ of the mesentery.

*History, Pathology, etc., of Trichinosis.**—Zenker's case, fully described and analyzed in 1859, fixed the diagnosis of the disease, and soon other cases were recognized, and former anomalous cases which had been looked upon as typhous or blood intoxication, were recognized as cases of trichinosis.

In 1860, at Karbach, three persons sickened from the use of trichinous meat. A case occurred at Detmold, which was thought to be a case of poisoning. The first endemic broke out at Plaven; was observed and described by Bœhler and Kœnigsdœrfer, and here the diagnosis was settled by finding the muscular trichinæ through the means of harpoons. Nineteen or twenty persons sickened, of whom one died. Since then the Kingdom of Saxony, as well as the Province of Saxony, seem to have been the focus of infection. A case of trichinosis was also carefully observed and described by Friedreich, of Heidelberg. In 1863, a slight endemic occurred at Rugen. About this time Langenbeck's case attracted much attention. He extirpated a cancroid tumor from the occipital muscle, and in the tumor, when opened, *trichinæ* were found in large numbers. The origin of this trichinosis was traced

---

* Dr. Roll's lecture before the Society of Physicians, Vienna.

back to a dinner party, at which the patient was present, nine years previously, and after which all the participants had sickened. At the time, the landlord had been accused of poisoning the wine. But this subsequent operation brought the whole event to a clear light. During the second half of October, 1863, a trichinæ epidemic broke out at Hettstadt, one of the most extensive and severest which ever occurred. Of one hundred and fifty-nine cases, twenty died. This endemic has been minutely detailed, and it was remarkable, that while the compositors in the printing establishment at Hettstadt were engaged with Rupperch't's brochure on the subject, a second epidemic broke out among them. Since then cases of trichinosis were observed at Leipzig, Berlin, Guedlinburgh, mostly traceable to the use of *raw* sausage meat. In Hamburg a sailor, coming from Valparaiso, sickened of the disease and died. From British India a case of trichinosis has been reported. At the close of 1865 the disease broke out at Hadersleben. The mortality here reached a very high figure. Of three hundred and three cases, ninety died; and twenty are yet reported to be down beyond hope of recovery. A remarkable feature in this endemic is, that fatal cases occurred as early as during the first week of sickening, while ordinarily, they do not occur before the third week. This is owing probably to peritonitis, and intense lesion of the intestines, from the large number of trichinæ introduced. At the commencement of this year the endemic broke out at Wecksdorf, on the frontier of Saxony and Bohemia. Trichinosis was here demonstrated by harpooning. Kuchenmeister discovered it in the case of a woman, Klob in that of a man.

The perforation of the intestines, and final perforation of the peritoneum, cause irritation of the intestines and peritoneum, which lead to inflammation. A careful study of Furstenberg has led to the reason why these inflammations have been so differently estimated by various observers. In some animals on which experiments were made to determine the migration of trichinæ, and which died, the inflammation was found so slight, that it could not be considered as the cause of death; in such cases it is not improbable that when the intestinal walls were perforated lymphatics may have been opened into, through which morbid substances were conveyed into the current of the blood, causing death. The peritonitis was generally marked by the presence of a certain quantity of reddish, turbid liquid in the peritoneal cavity, and trichinæ embryos were found in this exravasation. Upon the free surface of the peritoneum, embryos were always found in greater or less number.

*Symptomatology.*—Regarding symptomatology Dr. Roll divides the disease into three stages: 1st, stage of immigration; 2d, stage of digression; 3d, stage of regression. Death may occur in the first stage from peritonitis and enteritis; in the latter stages, death generally ensues from metastatic pneumonia, in consequence of venous thrombosis. The pain in the muscles, and the flexed position of the extremities are characteristic, probably because the trichinæ possess a predilection for the flexors; furthermore, œdema in consequence of disturbances in the circulation.

*Treatment.*—Regarding *therapeutics*, the picrimic salts, oil of turpentine, benzine, and also various anthelmintics, have been found useless. The best effects were derived from cathartics, at the commencement, especially calomel, in large doses, up to a scruple; later, quinine and iron.

*Etiology.*—In regard to the *etiology* of trichinosis there is no doubt. It is positively known that the infection takes place by trichinous pork. To Kuhn, President of the Veterinary institute at Halle, several questions were submitted, viz:

1st. Are there characteristic signs by which the trichinosis hog may be detected? The results of feeding hogs on trichinæ were, that the appearances of trichinosis in swine were enteritis, colic, and slight paralytic symptoms of the posterior extremities; these symptoms are, however, by no means characteristic, and occur in other diseases to which the animal is subject. 2d. Kuhn has determined by observation, that swine of every age and race, from the common hog to the best improved stock, may become trichinous. 3d. It was shown that trichinosis did not interfere with the process of fattening. Trichinæ were found in an animal which had been sold for seventy dollars. 4th. By harpooning, the diagnosis of trichinosis may best be ascertained. Regarding the relative frequency of trichinæ in the various muscles, Kuhn has given a scale. They occur most frequently in the diaphragm, in the muscles of the loin, then in the muscles of the shoulder; less frequently in the tongue, the larynx, etc. It is remarkable that they never occur in the involuntary muscles, in the heart, or in adipose tissue.

*Preventive Measures.*—Regarding prophylaxis, the most careful cleanliness in raising stock cannot be too urgently recommended, although it can readily be seen how difficult this is with an animal which is as omniverous as the hog. Feeding powdered anthracite has not been found to be of prophylactic value.

Dr. Roll discusses the measures of sanitary police which government should employ to protect the people against the trichinæ. As it is well known that the hog is the only animal from which trichinæ are imparted to man, there are three means to protect against the danger of the disease.

1st. To completely forbid the consumption of pork. 2d. To admit only such pork for consumption as is positively known to be free of trichinæ. 3d. To give to pork a preparation in cooking, by which the trichinæ which may be present are surely killed, and hence rendered inert.

Regarding the first means it may readily be seen that such a measure, for many reasons, is impracticable, as pork forms the most frequent animal food of the population. Regarding the second, a general microscopical inspection of the meat, under supervision of the government, as advocated by Virchow, is hardly practicable. Thus, for instance, in Vienna, where annually about 90,000 hogs are killed, it would demand an extraordinary force of examiners to carry the measure through; and the difficulty is yet increased, if we consider that of 20,000 hogs hardly one is found trichinous, even in countries where this disease occurs most frequently. An examiner, who for months in vain searches for an object, becomes tired out; and the correctness of such inspections could hardly be relied upon, aside from the fact that we do not yet possess abattoirs for hogs, and the inspectors would have to visit the houses of butchers, hotel-keepers, and private dwellings, to carry out the method. An imperative inspection of pork is hence considered impracticable.

But one method remains to ward off the danger of infection—by proper cooking, or preparation of the meat. It can be assumed with certainty, that a temperature of 60° R. kills the trichinæ. In cooking large pieces of pork for a short time, and in rapidly roasting chops, or cutlets, the interior of the meat generally does not reach more than 84° R. The great object, hence, in the preparation of pork, is thorough and complete cooking—rather too long than too little, and slowly rather than rapidly.

